# Supplementary material for: Causal interplay between lactose intolerance and gut microbiota: a combined bidirectional Mendelian randomization and in vivo validation study
Source: Front Nutr. 2026 Jun 1;13:1803337. doi: 10.3389/fnut.2026.1803337 (PMC13265576; doi:10.3389/fnut.2026.1803337)
Supplement: Supplementary file 1 [file Data_Sheet_1.zip › supplementary materials/Forward/forest plot/ebi-a-GCST90027697.finngen_R12_E4_LACTONAS.pdf]

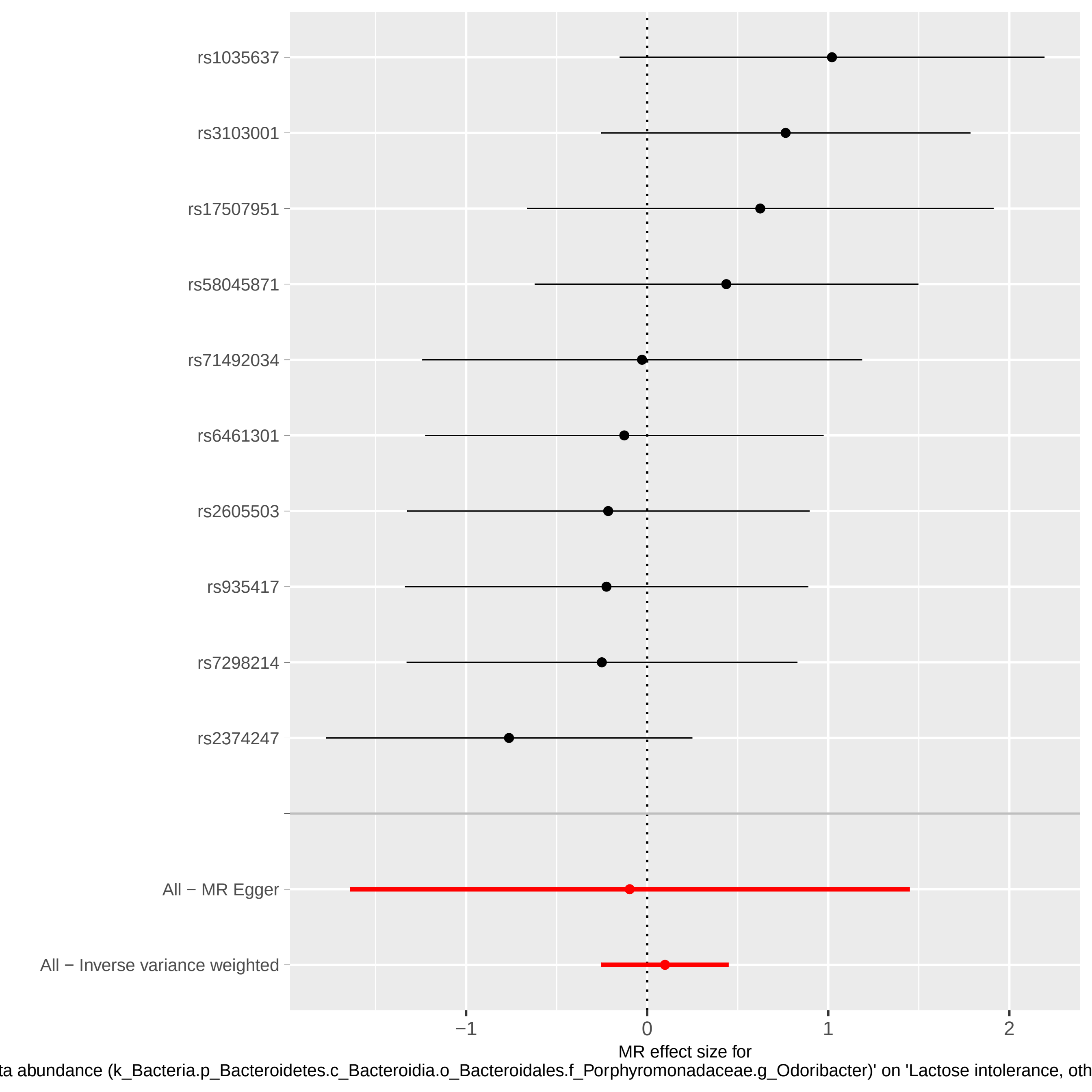

rs1035637
rs3103001
rs17507951
rs58045871
rs71492034
rs6461301
rs2605503
rs935417
rs7298214
rs2374247
All − MR Egger
All − Inverse variance weighted
−1
0
1
2
MR effect size for
ta abundance (k_Bacteria.p_Bacteroidetes.c_Bacteroidia.o_Bacteroidales.f_Porphyromonadaceae.g_Odoribacter)' on 'Lactose intolerance, oth
